# Olinciguat, an Oral sGC Stimulator, Exhibits Diverse Pharmacology Across Preclinical Models of Cardiovascular, Metabolic, Renal, and Inflammatory Disease

**DOI:** 10.3389/fphar.2020.00419

**Published:** 2020-04-08

**Authors:** Daniel P. Zimmer, Courtney M. Shea, Jenny V. Tobin, Boris Tchernychev, Peter Germano, Kristie Sykes, Ali R. Banijamali, Sarah Jacobson, Sylvie G. Bernier, Renee Sarno, Andrew Carvalho, Yueh-tyng Chien, Regina Graul, Emmanuel S. Buys, Juli E. Jones, James D. Wakefield, Gavrielle M. Price, Jennifer G. Chickering, G. Todd Milne, Mark G. Currie, Jaime L. Masferrer

**Affiliations:** ^1^Research and Development, Cyclerion Therapeutics, Cambridge, MA, United States; ^2^Research and Development, Ironwood Pharmaceuticals, Boston, MA, United States

**Keywords:** olinciguat, soluble guanylate cyclase, cGMP, nitric oxide, cardioprotective, renoprotective, vascular inflammation

## Abstract

Nitric oxide (NO)-soluble guanylate cyclase (sGC)-cyclic 3',5' GMP (cGMP) signaling plays a central role in regulation of diverse processes including smooth muscle relaxation, inflammation, and fibrosis. sGC is activated by the short-lived physiologic mediator NO. sGC stimulators are small-molecule compounds that directly bind to sGC to enhance NO-mediated cGMP signaling. Olinciguat, (R)-3,3,3-trifluoro-2-(((5-fluoro-2-(1-(2-fluorobenzyl)-5-(isoxazol-3-yl)-1H-pyrazol-3-yl)pyrimidin-4-yl)amino)methyl)-2-hydroxypropanamide, is a new sGC stimulator currently in Phase 2 clinical development. To understand the potential clinical utility of olinciguat, we studied its pharmacokinetics, tissue distribution, and pharmacologic effects in preclinical models. Olinciguat relaxed human vascular smooth muscle and was a potent inhibitor of vascular smooth muscle proliferation *in vitro*. These antiproliferative effects were potentiated by the phosphodiesterase 5 inhibitor tadalafil, which did not inhibit vascular smooth muscle proliferation on its own. Olinciguat was orally bioavailable and predominantly cleared by the liver in rats. In a rat whole body autoradiography study, olinciguat-derived radioactivity in most tissues was comparable to plasma levels, indicating a balanced distribution between vascular and extravascular compartments. Olinciguat was explored in rodent models to study its effects on the vasculature, the heart, the kidneys, metabolism, and inflammation. Olinciguat reduced blood pressure in normotensive and hypertensive rats. Olinciguat was cardioprotective in the Dahl rat salt-sensitive hypertensive heart failure model. In the rat ZSF1 model of diabetic nephropathy and metabolic syndrome, olinciguat was renoprotective and associated with lower circulating glucose, cholesterol, and triglycerides. In a mouse TNFα-induced inflammation model, olinciguat treatment was associated with lower levels of endothelial and leukocyte-derived soluble adhesion molecules. The pharmacological features of olinciguat suggest that it may have broad therapeutic potential and that it may be suited for diseases that have both vascular and extravascular pathologies.

## Introduction

Soluble guanylate cyclase (sGC) is a major receptor for nitric oxide (NO) and a key signal-transduction enzyme in the NO-cyclic guanosine 3',5'-monophosphate (cGMP) signaling pathway (Derbyshire and Marletta, 2012). The NO-sGC-cGMP pathway is an established target for cardiovascular drugs (e.g., nitroglycerin for angina and acute heart failure, sodium nitroprusside for hypertension, isosorbide mononitrate for chronic heart failure, phosphodiesterase 5[PDE5] inhibitors for pulmonary arterial hypertension) and non-cardiovascular drugs (e.g., PDE5 inhibitors for erectile dysfunction and benign prostatic hyperplasia).

sGC stimulators are a class of small molecule drugs that also target the NO-sGC-cGMP pathway (Sandner et al., 2018). The sGC stimulator riociguat is approved for the treatment of two rare and serious forms of pulmonary hypertension: pulmonary arterial hypertension (PAH) and chronic thromboembolic pulmonary hypertension (Ghofrani et al., 2013a; Ghofrani et al., 2013b). sGC stimulators have also shown benefit across a broad range of preclinical disease models including cardiac and kidney disease, pulmonary and skin fibrosis, metabolic disorders, and liver disease [reviewed in (Sandner et al., 2018)].

The sGC stimulator mechanism of action is unique in its ability to specifically enhance NO-driven cGMP signaling by acting in synergy with endogenous NO, a short-lived (half-life 5–10 s) paracrine signaling molecule (Stasch and Hobbs, 2009). In contrast, PDE5 inhibitors are not specific to NO-derived cGMP because they inhibit degradation of cGMP arising from both sGC and particulate guanylate cyclases. NO donor drugs and sGC activators also act on the NO-sGC pathway but their action is independent of endogenous NO production and thus can overwhelm the endogenous NO signaling, which is local and transient in nature.

The beneficial effects of sGC stimulators observed across a variety of disease models may be explained by the fact that sGC is expressed in a broad range of cells and tissues (Budworth et al., 1999). The product of sGC activity, cGMP, is a central cellular signaling molecule that plays a role in regulating important cellular processes including smooth muscle relaxation and proliferation, inflammation, and fibrosis (Buys et al., 2018).

Given the important but different roles that sGC plays in the vasculature and in extravascular tissues, controlling compound distribution in order to target pharmacologic activity to these sites may be a promising strategy for developing disease-specific sGC stimulators. For instance, a compound with pharmacological activity in the vasculature may provide greater benefit in diseases where effects on blood flow and vascular inflammation are desirable. Therefore, to understand the potential clinical utility of a new sGC stimulator, it is important to explore its pharmacokinetics, tissue distribution, as well as vascular versus extravascular pharmacologic effects in preclinical models.

Here we report for the first time the pharmacologic properties of the novel, clinical-stage sGC stimulator olinciguat. We show its pharmacologic activity *in vitro*, including in a human whole cell sGC activity assay and vascular smooth muscle relaxation and proliferation assays. We characterize its pharmacokinetics, mass balance, and tissue distribution in rats, and compare tissue levels to plasma levels. Finally, we present the pharmacology of olinciguat in several preclinical rodent models. We report effects of olinciguat on blood pressure in normotensive and hypertensive rats; inflammation in a mouse model; and cardiac, renal, and metabolic effects in a rat model of metabolic syndrome and diabetic kidney disease.

## Materials and Methods

### Compounds, Materials, and Diets

Olinciguat, (R)-3,3,3-trifluoro-2-(((5-fluoro-2-(1-(2-fluorobenzyl)-5-(isoxazol-3-yl)-1H-pyrazol-3-yl)pyrimidin-4-yl)amino)methyl)-2-hydroxypropanamide, was synthesized by Cyclerion Therapeutics. Enalapril (#E6888), sodium nitroprusside (SNP, #71778), and 3-isobutyl-1-methylxanthine (IBMX, #I5879) were from Sigma. Tadalafil (#14024) was from Cayman Chemical. Diethylenetriamine NONOate (DETA, #ALX-430-014) was from Enzo Life Sciences. TNFα (#410-MT-50), mouse sICAM-1 ELISA kit (#MIC100), mouse sP-selectin ELISA kit (#MPS00), and mouse sE-selectin ELISA kit (#MES00) were from RnD Systems. The mouse sL-selectin ELISA kit (#ab155448) was from Abcam. PicoLab Rodent Diet 20 chow (LabDiet) was used in the Wistar and SHR hemodynamic studies.

Open Standard Diet (Research Diets, Inc., New Brunswick, NJ, #D11112201) was used as the normal salt (NS) diet in the Dahl salt-sensitive (DSS) rat model, the post-myocardial infarction (MI) model, and the mouse vascular inflammation model. The high salt (HS) diet in the DSS model consisted of Open Standard Diet formulated with 8% NaCl. Purina 5008 normal rodent chow (Purina, #C1300) was used in the ZSF1 study. For studies in which olinciguat was provided in chow, olinciguat was formulated at 83 mg/kg in chow which targeted a 10 mg/kg/day dose and 250 mg/kg in chow targeted a 30 mg/kg/day dose. In the ZSF1 study, enalapril was dissolved in the drinking water.

### Animals

All animals studied were housed in animal facilities accredited by the Association for Assessment and Accreditation of Laboratory Animal Care; all animal-use protocols were reviewed and approved by the Institutional Animal Care and Use Committee prior to commencement. Rats studied in the DSS and post-MI model and mice studied in the acute inflammation model were from Envigo. Rats in the hemodynamic and ZSF1 studies were from Charles River Laboratories.

### Human Tissues

Human subcutaneous resistance arteries studied in vascular tissue relaxation experiments were obtained from three donors with proper authorization and full ethical approval (ReproCELL Europe Ltd., Beltsville, MD). The collection and use of tissue was approved by the West of Scotland Research Ethics Committee (12/WS/0069) and all participating donors provided informed written consent prior to the surgical procedure.

### sGC Whole Cell Assay

The human embryonic kidney (HEK)-293 whole cell assay was performed as described (Tobin et al., 2018). Briefly, HEK-293 cells (ATCC, Manassas, VA, #CRL-1573) were maintained in Dulbecco's modified Eagle's medium (DMEM) containing serum and antibiotics. Assays were performed in 384-well plates at 37°C by preincubating with 0.5 mM 3-isobutyl-1-methylxanthine (IBMX) in Hanks' Balanced Salt Solution (HBSS) containing calcium and magnesium for 15 min, then adding olinciguat with or without the NO donor diethylenetriamine NONOate (DETA), followed by a 20-min incubation. Cells were lysed with ice-cold 10% acetic acid then centrifuged, and cGMP levels in supernatants were determined by reverse-phase liquid chromatography with tandem mass spectrometry (LC-MS/MS). Concentration-response data were fit using a four-parameter fit (log(agonist) vs response – variable slope) using GraphPad Prism v.7.

### Human Vascular Smooth Muscle Relaxation

The effect of olinciguat on relaxation of contracted human subcutaneous resistance arteries was determined according to methods described in (Tobin et al., 2018). In brief, human subcutaneous resistance arteries were obtained from three donors (females ages 27–51) undergoing elective surgeries. Arteries were mounted in a tissue bath and precontracted with the thromboxane mimetic, U46619 (100 nM). Only tissues containing functional endothelium, as determined by relaxation in response to acetylcholine (10 µM), were used. A cumulative concentration-response curve (0.1 to 10 µM) was generated for olinciguat, followed by addition of the NO donor sodium nitroprusside (100 μM). Percent relaxation at each olinciguat concentration was determined relative to the U46619-mediated contraction. Using GraphPad Prism v.7, a sigmoidal dose-response (variable slope) curve was fit with the bottom value constrained to zero to determine the EC_50_.

### Rat Aorta Vascular Smooth Muscle Cell Proliferation

We explored the concentration response of olinciguat, tadalafil, and olinciguat in the presence of 1 μM tadalafil on rat aorta vascular smooth muscle proliferation, Tadalafil was used because it is a once-daily PDE5 inhibitor that, like the three-times-daily PDE5 inhibitor sildenafil, is approved for treatment of PAH and ED. Vascular smooth muscle cells (VSMC) from rat aorta (Lonza, R-ASM-580) were plated into a 96-well plate at 15,000 cells per well in DMEM:F12 containing 20% FBS and GA-1000 ([Gentamicin, Amphotericin B], Lonza, CC-40863) for 24 h at 37°C. Medium was removed and cells were washed once with 100 μl DMEM:F12 before adding 100 μl of DMEM:F12 and incubating for 24 h to induce quiescence. Cells were then cultured for 24 h in DMEM:F12 supplemented with 5% FBS, in the presence of test articles (vehicle, olinciguat, tadalafil) with 10 µM DETA. In the last 6 h, 0.2 μCi of ^3^H-thymidine (American Radiolabeled Chemicals, ART 0178A) was added to each well to measure DNA synthesis by thymidine incorporation. Cells were washed once with HBSS and then trypsinized and transferred into FB filter plates (Millipore, MADVNOB). A rapid-filtration technique using a filtration apparatus (Millipore, MAVM0960R) with vacuum aspiration was used to harvest and rinse labeled cells (6× with 0.2 ml of chilled water). The radioactivity bound to the filters was counted with 80 µl of liquid scintillant (PerkinElmer, UltraGold MV, 6013159) in a scintillation counter (Perkin Elmer Microbeta instrument).

### Pharmacokinetics

Male (n=12, 2 groups of 6) and female (n=12, 2 groups of 6) Sprague-Dawley rats (275–300 g) with indwelling jugular vein cannulae were fasted overnight prior to dosing. Animals were dosed either intravenously (i.v.) *via* bolus injection through a percutaneous catheter into a lateral tail vein or orally (p.o.) *via* gavage. Olinciguat was formulated in 60% polyethylene glycol (PEG) 400/40% water at 0.3 mg/ml (0.3 mg/kg) for i.v. dosing, and at 1 mg/ml (1 mg/kg) in 100% PEG 400 for p.o. dosing. Chow was returned to animals 4 h postdose.

Blood from i.v. and p.o. dosed rats was collected at 16 timepoints (2 groups of rats, 8 timepoints per group) over 24 h. Samples were collected into potassium ethylenediamine tetraacetic acid (K_2_EDTA) tubes, centrifuged (3,500 rpm for 10 min) to yield plasma. In the pharmacokinetic studies and in all *in vivo* studies, olinciguat plasma concentrations were determined by LC-MS/MS. Pharmacokinetic (PK) parameters were calculated by noncompartmental analysis using sparse sampling in Phoenix^®^ WinNonLin^®^ v.6.3 (Certara, Princeton, NJ).

### Plasma Protein Binding

Concentrated (10 mM) DMSO stock solutions of olinciguat were diluted to 1 and 10 μM in 2.5 ml of rat (male and female Sprague Dawley) and human plasma. Concentrated [^14^C]-labeled olinciguat (1 μl,1 mCi/ml stock concentration) was spiked into each sample and mixed by vortexing. Triplicate 500 μl aliquots of plasma containing [^14^C]-labeled olinciguat were transferred to a Rapid Equilibrium Dialysis system and dialyzed (molecular weight cut-off ~8000 Da) against 500 μl of phosphate-buffered saline (PBS) at room temperature for 18 h. Radioactivity of [^14^C]-labeled olinciguat in PBS and plasma samples was measured using liquid scintillation counting.

### Mass Balance/Excretion

[^14^C]-olinciguat, uniformly labeled with [^14^C] in the fluorobenzyl ring and having a specific activity of 110 mCi/mmol, was synthesized at American Radiolabeled Chemicals, Inc. (St. Louis, MO). The radiochemical purity was determined to be >99%. Four male Sprague-Dawley rats (approximately 8 weeks old) received a single p.o. dose of 3 mg/kg [^14^C]-olinciguat (specific activity 26.6 µCi/mg) in PEG 400:water 60:40 v/v (dosing volume ≤5 ml/kg). Using a single metabolism cage (Nalgene) for each rat, urine and feces were collected for 7 days at 24-h intervals, and cages were rinsed daily. Food was available ad libitum throughout the study, except for the overnight fasting period (16–18 h) before and 4 h after olinciguat administration. Deionized water was available ad libitum. After 7 days, tissues were collected. Urine, feces, liver, kidneys, and cage washes were analyzed to determine material balance.

### Quantitative Whole-Body Autoradiography

Quantitative whole-body autoradiography (QWBA) assays were performed at QPS, LLC (Newark, DE) as described previously (Solon et al., 2002). Male Long-Evans (LE) rats (218–262 g, n=11, Hilltop Lab Animals, Inc., Scottdale, PA) were studied. All rats were administered a single p.o. dose of [^14^C]-olinciguat at a target dose of 3 mg/kg and approximately 200 µCi/kg formulated in PEG 400 in water (60:40 v/v). Immediately prior to euthanasia, blood was obtained *via* cardiac puncture for plasma radioactivity determinations by liquid scintillation counting. At each timepoint (1, 2, 4, 8, 12, 24, 48, 72, 96, 168, and 504 h post-dose), one rat was euthanized by immersion in a hexane/solid carbon dioxide bath and frozen for at least 15 min for QWBA analysis. Each frozen rat carcass was embedded in a 2% carboxymethylcellulose matrix with a microtome stage at −20°C. Concentrations of radioactivity were used to determine the concentration of compound equivalents in each tissue and in plasma using the Specific Activity of 66.7 µCi/mg of [^14^C]-olinciguat. Whole body sections for each rat were mounted on a cardboard backing, covered with thin plastic wrap, and exposed along with calibration standards of [^14^C]-glucose at 10 different concentrations (0.00096 to 7.8 µCi/g) to a ^14^C-sensitive phosphor imaging plate (Fuji Biomedical, Stamford, CT). Imaging plates were scanned using the Typhoon 9410 image acquisition system (GE/Molecular Dynamics, Sunnyvale, CA, USA). Radioactivity concentration in tissues was quantified by image densitometry using MCID image analysis software (v. 7.0, Interfocus Imaging Ltd) and a standard curve constructed from the integrated response (molecular dynamics counts/mm^2^) and the nominal concentrations of [^14^C]-glucose standards. Tissue concentration data were obtained for virtually all tissues (see **Supplemental Materials**). The concentrations of radioactivity were expressed as the microgram equivalents of [^14^C]-olinciguat per gram of sample. An upper and lower limit of quantification, determined by using the radioactive concentration of the highest and lowest calibration standards divided by the specific activity of the test article in formulation, was applied to the data. Phoenix^®^ WinNonLin^®^ v.6.3 was used to determine PK parameters, including maximal concentration (C_max_) and area under the curve of all observed points (AUC_all_). Tissue:plasma AUC_all_ ratios were determined.

### *In Vivo* Models

For all *in vivo* pharmacology models described below, animals were housed under controlled conditions of temperature (22.2 ± 4.4°C) and relative humidity (30–70%) in a 12-h light-dark cycle. All animals were acclimated to the facility for at least 3 days prior to study initiation. Rats and mice had ad libitum access to water and food, except during fasting periods.

#### Measurement of Arterial Hemodynamics by Telemetry

The Dataquest A.R.T.™ acquisition and analysis system (Data Sciences International [DSI], St. Paul, MN) was employed to monitor and analyze hemodynamic data from conscious, freely moving rats surgically implanted with a telemetry pressure transmitter (PA-C40). Telemetry transmitter implantation was performed on rats under sterile conditions. Rats were anesthetized with isoflurane and body temperature was maintained with a heating pad during surgery. A laparotomy was performed to expose the abdominal aorta. The catheter tip of the telemetry transmitter was inserted into the abdominal aorta and secured with a 5-0 silk suture (Ethicon, Inc., Somerville, NJ). The abdominal incision was closed with uninterrupted suture (4-0 silk, Ethicon, Inc., Somerville, NJ) and the telemetry transmitter was placed in the abdominal cavity and secured to the abdominal wall. Approximately 100 µl of 0.25% marcaine was applied directly to the closed abdominal wall, and the skin was then closed with suture (4-0 Vicryl^®^ absorbable, Ethicon, Inc., Somerville, NJ). Buprenorphine (0.05 mg/kg/day, subcutaneous [s.c.]) was administered immediately after the surgery for postoperative pain relief. After recovery from anesthesia, rats were returned to their home cages, placed on DSI receivers, and allowed a 5- to 14-day recovery period. Arterial hemodynamics were measured continuously and recorded throughout the study. For acute studies, analysis was based on hourly averages, for chronic studies, analysis was based on daily or weekly averages.

#### Hemodynamics in Normotensive Wistar and Spontaneously Hypertensive Rats

Spontaneously hypertensive rats (SHRs) (n=6, male, 230–250 g, 14 weeks of age) and Wistar rats (n=6, male, 230–250 g, 12 weeks of age) were implanted with telemetry transmitters as described above. Olinciguat was formulated as a suspension in a vehicle containing 0.5% methylcellulose and 0.2% Tween. Rats were p.o. dosed with a dosing volume of 5 ml/kg. In a weekly-dose escalation design, rats were administered vehicle (week 1), olinciguat 1 mg/kg (week 2), 3 mg/kg (week 3), and 10 mg/kg (week 4). For each dose level, rats were dosed once daily (QD) for 4 days. Using plasma from tail vein, olinciguat plasma concentrations were determined at 1, 6, and 24 h post-dose on day 4 and reported as the mean from Wistar (n=3) and SHR (n=3). Hemodynamics were recorded hourly during a 24-h baseline period and over the first 3 days of dosing.

Maximum change from baseline of mean arterial pressure (MAP) was calculated using the 24-h pre-first dose average subtracted from the lowest MAP value recorded within 2 h following dosing. The 1-h post-dose change from baseline heart rate (HR) was calculated using the 3-h pre-first dose average subtracted from the 1-h post-dose average.

#### Dahl Salt-Sensitive Rat Model

Male DSS rats (n = 48, 230–300 g) were implanted with telemetry transmitters as described above. During the 2-week pretreatment period and the 6-week treatment period, rats were either fed a NS diet (0.3% NaCl, n=8) or a HS diet (8% NaCl). During the treatment period, one group of HS rats was continued on HS diet (HS, n=8) and a second group of HS rats was switched to a HS diet containing olinciguat targeting the dose equivalent of 10 mg/kg/day (OLI10, n=8). At the end of the 6-week treatment period, body weight was measured, urine was collected over a 24-h period using a metabolic cage for urinalysis, and blood samples were collected for plasma biomarker analyses. At the end of the study, rats were anesthetized, and whole bodies were perfused *via* the left ventricle with heparinized PBS (1 U/ml heparin) containing IBMX (1mM). Organs were collected and weighed. All organ weights were normalized to body weight. After weighing the heart, the right ventricular free wall was removed, and the left ventricular free wall plus left ventricular septum (LV+S) were weighed.

#### Rat Post-myocardial Infarction Model

Male Sprague-Dawley rats (n = 24, 270–330 g) were studied. MI was induced by permanent ligation of the left anterior descending (LAD) coronary artery. Briefly, after anesthesia with 5% isoflurane, rats were intubated and ventilated using a small animal ventilator (Model 683, Harvard Apparatus). Surgery was performed under sterile conditions. Briefly, the heart was exposed through left thoracotomy, the pericardium was incised, and the LAD coronary artery was ligated with 4-0 silk suture (Ethicon, Inc.). The lungs were inflated, and the chest wall was closed in three layers. Approximately 100 µl of 0.25% marcaine was applied directly to the edge of the skin incision. Long-acting buprenorphine (1 mg/kg/day, s.c.) was administered after the surgery for postoperative pain relief. Sham-operated rats underwent the same procedure without ligation of the LAD.

Treatment was initiated within 24 h following surgery. The Sham and MI control groups were maintained on chow for the entire 8-week treatment period. Rats in the MI + olinciguat group received olinciguat in chow at the targeted dose of 30 mg/kg/day for the entire 8-week treatment period. Ejection fraction (EF) was measured at week 8 by echocardiography using the long axis view of the left ventricle in B-mode.

#### ZSF1 Rat Model

Lean (n=8, 370–420 g, 15 weeks) and obese (n=32, 500–630 g, 15 weeks) male ZSF1 rats were studied. Rats were treated with enalapril to reflect standard of care drug treatment for patients with diabetic kidney disease. Dose modeling based on allometric scaling relationships estimated that a 10 mg/day enalapril dose in humans (within usual dose range of 10–40 mg based on prescribing information) corresponds to 1 mg/kg/day in rats (Nair and Jacob, 2016). To measure hemodynamics in freely moving animals, ZSF1 obese rats were implanted with telemetry transmitters (HD-S10, Data Sciences International) at 9–10 weeks of age, as described above.

The study included a 10-day pretreatment period and a 10-week treatment period. There were five groups of rats in the study. The lean ZSF1 control group (lean control, n=8) and one obese group (obese control, n=8) were fed normal chow and drinking water throughout the 10-day pretreatment period and the 10-week treatment period. Three groups of obese rats were treated with enalapril in drinking water (equivalent to 3 mg/kg/day) throughout the 10-day pretreatment period and the 10-week treatment period. During the treatment period, these enalapril-treated obese rats were administered normal chow (ENP, n=8) or chow containing olinciguat targeting the dose of 10 mg/kg/day (ENP+OLI10, n=8) or 30 mg/kg/day (ENP+OLI30, n=8). Blood and 24-h urine samples were obtained from all animals at baseline (immediately prior to initiation of enalapril treatment), the end of the 10-day pretreatment period (Day 10) and at week 5 and week 10 of the treatment period. Plasma samples were obtained from all olinciguat-treated animals for measurement of olinciguat exposure at week 10. During week 9 of treatment, rats were fasted for 16 h overnight prior to determination of fasting blood glucose using the AlphaTRAK (Abbott) glucose monitoring system. Body weight was measured once every other week. At the end of the study, rats were anesthetized and perfused *via* the left ventricle with heparinized PBS (1 U/ml heparin) containing IBMX (1 mM). Organs were collected and weighed, and plasma was collected in EDTA tubes. For serum, blood was collected in serum separator tubes, maintained at room temperature for at least 30 min and centrifuged at 6,000 rpm for 10 min, and then stored at −80°C until analysis. Urine and serum samples were analyzed using the Randox Daytona Clinical Chemistry Analyzer (Randox, Kearneysville, WV) for urine protein, serum cholesterol and serum triglycerides. All measurements were performed in accordance with manufacturer's instructions.

For histology, one kidney from each rat was fixed in 10% formalin, then embedded in paraffin. Slides were prepared and stained with hematoxylin and eosin (H&E) and periodic-acid Schiff (PAS) for light microscopic analysis. Kidney sections were evaluated for the presence of glomerular pathology, tubular damage, interstitial inflammation, interstitial fibrosis, and vascular alterations. Severity-based scoring based on these evaluations was performed by a trained pathologist.

For the ZSF1 experiment, data are expressed as mean ± standard error of the mean (SEM).

#### Mouse Model of Vascular Inflammation

Male C57BL/6 mice (n=30, 22 g, 8 weeks) were studied. The TNFα/olinciguat group of mice (n=10) was administered 10 mg/kg olinciguat p.o., formulated in 0.1 ml 100% PEG, followed byintraperitoneal (i.p.) injection of 50 ng of mouse TNFα formulated in 0.5 ml pyrogen-free 0.9% NaCl solution one hour later. The TNFα/vehicle control group of mice (n=10) was treated with p.o. vehicle, followed by i.p. injection of 50 ng of mouse TNFα after one h. A naïve, non-treated control group of mice (n=10) was included in the study. Four hours after TNFα treatment, mice were euthanized and plasma from the inferior vena cava was collected in EDTA tubes and stored at −80°C until further analysis. Plasma levels of olinciguat were determined by LCMS. Plasma samples were diluted 1:300 for determination of sL-selectin, sP-selectin, sE-selectin, and sICAM-1 by ELISA.

### Statistics

All statistical analyses were performed in Graphpad Prism v.7. p-values <0.05 were considered significant.

In the vascular smooth muscle cell proliferation experiment, a comparison between the IC_50'_s for olinciguat alone vs oliniciguat in the presence of 1 µM tadalafil was performed by a two-tailed t-test using Log EC_50_ best fit values and SEs determined from a four-parameter nonlinear curve fit, assuming n=60 degrees of freedom.

In the hemodynamic study in Wistar and SHR, hourly MAP data were analyzed using a repeated measures two-way ANOVA, with time and treatment group as independent variables. Significance at each timepoint was determined as compared to the vehicle control using a Dunnett's post-hoc test. Hourly MAP values that were significantly different from the respective vehicle controls during the 3-day period of dosing and hemodynamic monitoring are shown.

In the DSS model, daily MAP (24-h average) data were analyzed using two-way ANOVA, with time and treatment group as independent variables. For each day, groups were compared to the HS group with a Dunnett's post-hoc test. Body weight, LV+S, cardiac weight, lung weight, kidney weight, plasma NT-proBNP, and 24-h urine protein excretion (UPE) data were analyzed by one-way ANOVA, followed by Dunnett's multiple comparison to the HS group.

In the post-MI study, data were analyzed by one-way ANOVA, followed by Dunnett's multiple comparison to the MI group.

In the ZSF1 study, change from baseline NT-proBNP, week 10 heart, kidney, and liver weight, urine volume, cholesterol, triglycerides, glucose, and renal histopathology sum score were analyzed by one-way ANOVA corrected for multiple comparisons using Tukey's method. Statistically significant differences vs the obese control and vs the ENP group are indicated in the results. MAP and UPE were analyzed by an ordinary two-way ANOVA with timepoint and treatment group as independent variables, followed by a Tukey's multiple comparisons test. Within each treatment group, significant differences vs baseline are indicated in the results. At week 10, statistically significant differences vs the obese control and vs the ENP control are also indicated in the results.

In the vascular inflammation model, data were analyzed by one-way ANOVA, followed by Dunnett's multiple comparison to the TNFα-vehicle control.

## Results

### Olinciguat Stimulated sGC in a Human Whole Cell Assay

**Figure 1:**
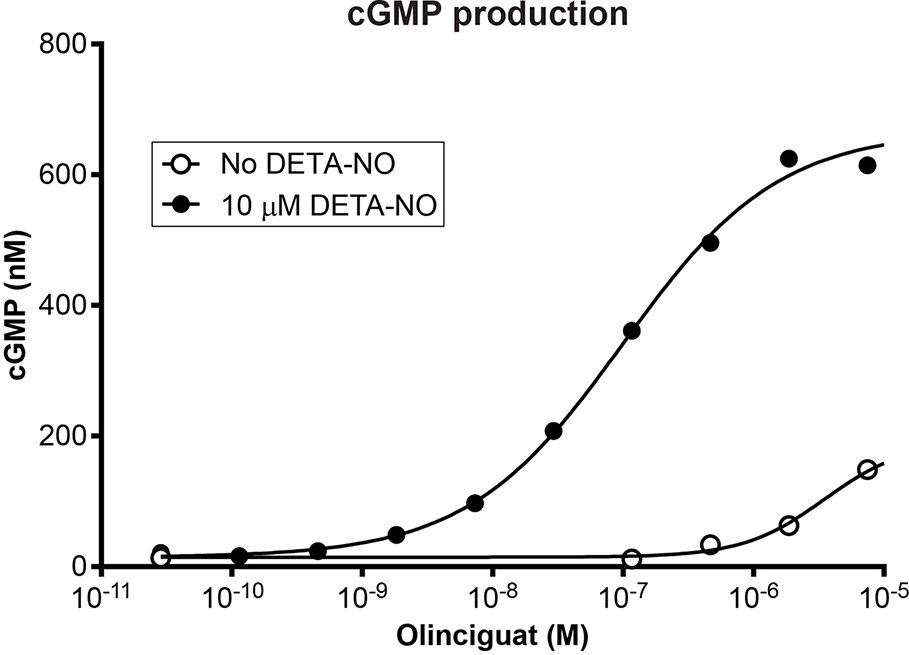
Representative olinciguat concentration response in HEK-293 cells, without NO and in the presence of 10 µM DETA.

Olinciguat is an sGC stimulator from a pyrazole-pyrimidine heterocyclic structural class of compounds. HEK-293 cells endogenously expressing sGC were treated with olinciguat in the absence or in the presence of the NO donor DETA. In the presence of 10 µM DETA, olinciguat stimulated cGMP production with EC_50_ = 73.8 nM (geometric mean of 14 separate experiments, 95% CI 42.7 to 105 nM). Olinciguat activated sGC synergistically with NO, as shown by the representative concentration response curves of olinciguat with 10 µM DETA, and without NO donor in **Figure 1**.

### Olinciguat Relaxed Human Vascular Smooth Muscle

**Figure 2:**
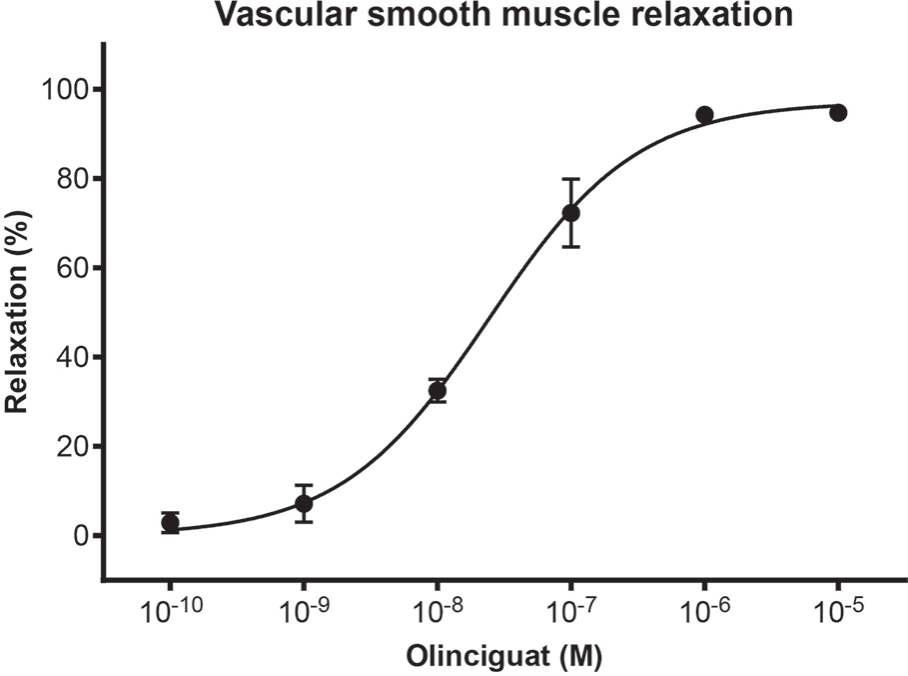
Concentration response of olinciguat in human subcutaneous resistance arteries pre-contracted with U46619 (100 nM). Percent relaxation is relative to the U46619-mediated contraction. Each data point represents the mean from assays in tissues from n=3 human donors, error bars represent the SEM.

In order to measure the effect of olinciguat on small vessels of human vasculature, human subcutaneous resistance arteries were pre-contracted with the thromboxane mimetic, U46619. Olinciguat caused a concentration-dependent relaxation with an EC_50_ of 24.3 nM (n=3, 95% CI 16 to 38 nM) (**Figure 2**). The mean relaxation at 10 μM was 94.8%.

### Olinciguat Inhibited Vascular Smooth Muscle Cell Proliferation *In Vitro*

**Figure 3:**
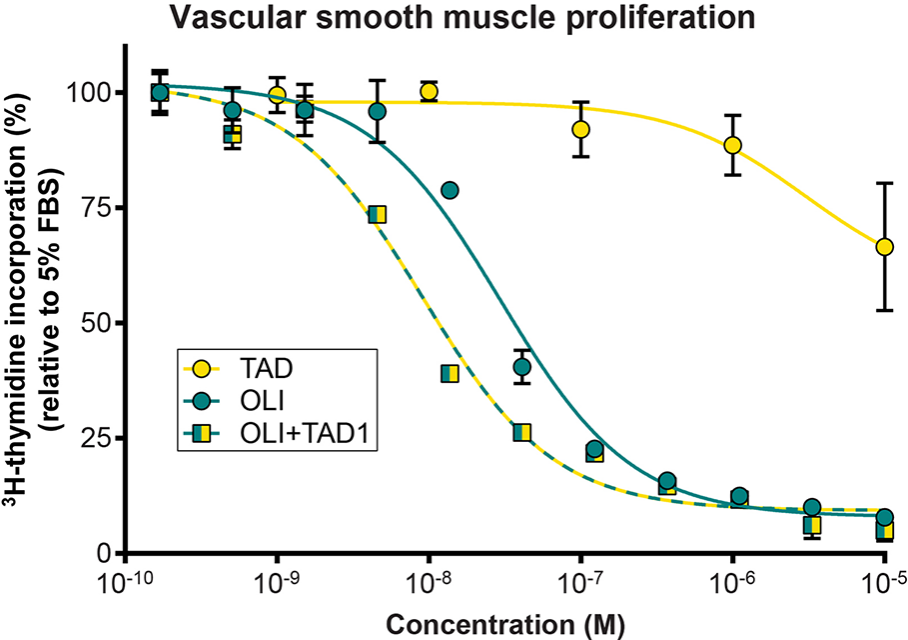
Effect of olinciguat on vascular smooth muscle cell proliferation. Concentration response of tadalafil alone (TAD), olinciguat alone (OLI), and olinciguat in the presence of 1 µM tadalafil (OLI + TAD1) on proliferation of VSMC from rat aorta, as measured by cellular ^3^H thymidine incorporation in response to 5% FBS. All assays included 10 µM DETA. Data are normalized to FBS control. Each data point represents the mean from n=3 independent assays, error bars represent the SEM. The IC_50_s for olinciguat alone vs in the presence of 1 µM tadalafil were significantly different from each other (p=2.4E-07).

We next studied the effects of olinciguat and the PDE5 inhibitor tadalafil, alone and in combination, on VSMC proliferation (**Figure 3**). Olinciguat alone inhibited rat aorta VSMC proliferation with an IC_50_ of 29.6 nM (95% CI 22.4 to 39 nM). In contrast, 10 µM tadalafil alone did not inhibit VSMC proliferation. However, 1 µM tadalafil, which corresponds to the C_max_ plasma concentration of tadalafil after 10 days of dosing at 20 mg QD in healthy volunteers (Forgue et al., 2006), potentiated the effect of olinciguat in this assay, shifting its potency by three-fold (mean IC_50_ = 9.1 nM, 95% CI 6.9 to 11.9 nM). The IC_50_s for olinciguat alone vs in the presence of 1 µM tadalafil were significantly different from each other. Similar results were observed for olinciguat alone and in combination with the PDE5 inhibitor sildenafil (data not shown).

### Pharmacokinetics and Mass Balance of Olinciguat

The PK profile of olinciguat was assessed in female and male Sprague Dawley rats. PK parameters are shown in **Table 1**. The steady-state volume of distribution (V_ss_) after i.v. administration was 0.9 L/kg (females) and 1.1 L/kg (males), indicating similar partitioning of olinciguat between the plasma and tissues of rats. Systemic clearance (CL) was low to moderate and lower in females (5.6 ml/min/kg) than in males (11.3 ml/min/kg). Following p.o. dosing, the time to reach maximum concentration (T_max_) for olinciguat was 3 h for females and 1 h for males with a maximum concentration (C_max_) of 198 ng/ml and 96.4 ng/ml, respectively. The oral t_1/2_ was 3.9 h (females) and 3.5 h (males), and the oral bioavailability was 45% in females and 36% in males.

**Table 1:** Pharmacokinetics of olinciguat in female and male Sprague Dawley rats.

PK Parameter		Female	Male
i.v.	V_ss_ (L/kg)	0.9	1.1
	CL (ml/min/kg)	5.6	11.3
p.o.	C_max_ (ng/ml)	198	96.4
	T_max_ (h)	3.0	1.0
	t_1/2_ (h)	3.9	3.5
Oral bioavailability		45%	36%

Pharmacokinetic parameters were calculated by noncompartmental analysis using sparse sampling. V_ss_, i.v. steady-state volume of distribution; CL, systemic clearance; C_max_, maximum plasma concentration after p.o. dosing; T_max_, sampling time of the maximum plasma concentration after p.o. dosing; t_1/2_, half-life during the apparent elimination phase after p.o. dosing.

Olinciguat was highly bound to rat (99.00 ± 0.26%) and human (98.01 ± 0.77%) plasma proteins. The fraction bound to plasma proteins was similar at olinciguat assay concentrations of 1 and 10 μM, and no gender difference was observed in olinciguat binding to rat plasma.

Following a single oral dose of 3 mg/kg [^14^C]-olinciguat, 95% of the administered dose radioactivity was recovered in feces and urine within the first 48 h after dosing. [^14^C]-olinciguat equivalents excreted in the feces and urine over 7 days were 95.3 and 5.7%, respectively, expressed as percent of the actual administered dose.

### Olinciguat Evenly Distributed to Plasma and Tissues

In a rat qWBA study [^14^C]-olinciguat-derived radioactivity was found in most tissues. Of the 44 tissues evaluated, those with tissue:plasma AUC_all_ ratios > 2.0 were: liver (50.3), small intestine (27.5), kidney cortex (13.6), kidney medulla (5.14), cecum (4.63), urinary bladder (2.62), adrenal gland (2.41), and esophagus (2.01). Most (28 of 44) tissues had tissue:plasma ratios between 0.5 and 2, including heart (0.84), lung (0.86), aorta (0.92), skeletal muscle (0.52), white adipose (0.67), and brown adipose (1.16). The remaining eight tissues had tissue:plasma AUC_all_ ratios ≤0.1, including all four measured regions of the brain, spinal cord, bone, and eye lens. The AUC_all_ blood-to-plasma ratio was 0.73.

### Olinciguat Reduced Blood Pressure in Normotensive Wistar Rats and SHR

**Figure 4:**
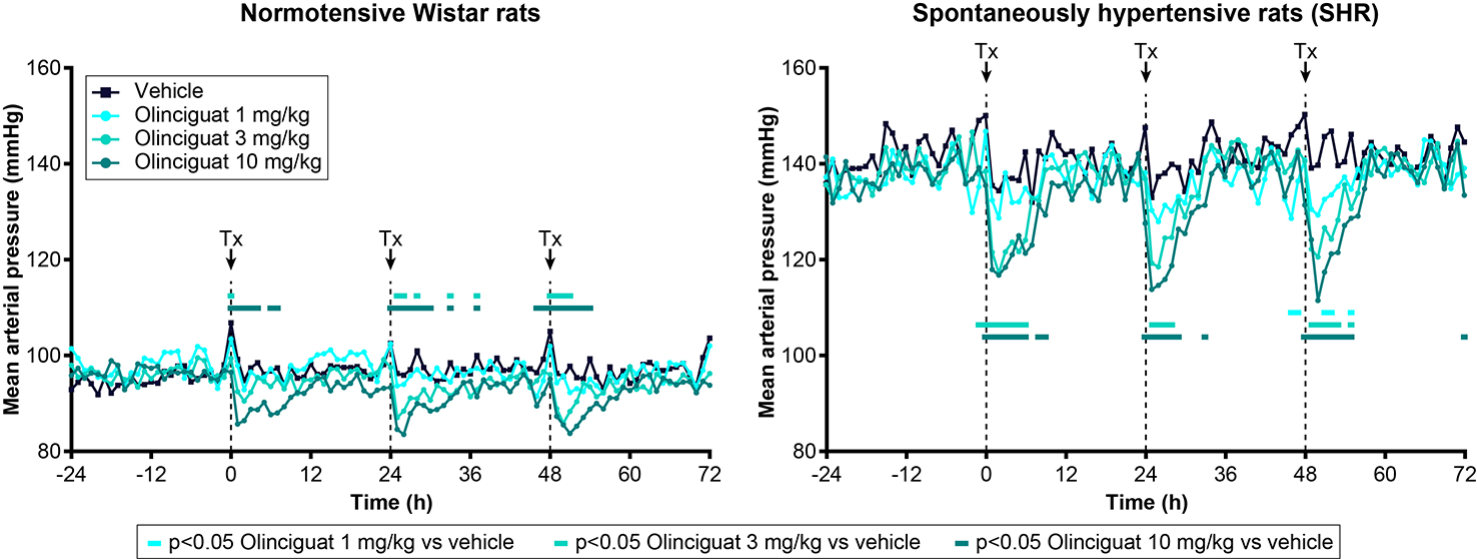
Effect of olinciguat dose on mean arterial pressure, measured by telemetry in freely moving normotensive Wistar and spontaneously hypertensive rats (SHR). Data are shown for baseline (−24 h to 0 h) and the first 3 days of dosing QD by oral gavage. Timing of dosing is indicated by dashed vertical lines at 0, 24, and 48 h. Data are plotted as means over time (n=6 per group), for simplicity, no error bars are included. Tx, treatment. For each treatment group, MAP measurements that were significantly (p < 0.05) lower than vehicle-treated rats are indicated by filled rectangles in the corresponding color.

Previous studies have shown that MAP in SHR is a robust, dose-responsive measure of target engagement for sGC stimulators (Mittendorf et al., 2009). Olinciguat reduced MAP in telemetered freely moving normotensive Wistar rats and SHR, when dosed QD by oral gavage (**Figure 4**). In SHR, MAP of rats treated with 3-mg/kg and 10-mg/kg olinciguat was significantly lower than vehicle-treated rats in the 6-h period immediately following dosing on all 3 days of dosing and hemodynamic monitoring. At the 1-mg/kg dose, MAP was only lower than vehicle-treated rats on the third day of dosing. In Wistar rats, MAP in the 10 mg/kg group was lower on all 3 days of dosing, MAP in the 3 mg/kg group was lower only on the second and third day of dosing, and MAP in the 1 mg/kg group was not significantly different from vehicle at any time.

At the 10 mg/kg dose (day 3), the maximum absolute change in MAP was −11 ± 3 mmHg in Wistar rats and −26 ± 2 mmHg in SHR. The 1-h post-dose change from baseline HR was 134 ± 10 BPM in Wistar rats and 86 ± 9 BPM in SHR (both at 10 mg/kg on day 3). In Wistar rats and SHR, the peak change in MAP at 10 mg/kg appeared consistent across the 3 days of dosing and hemodynamic monitoring. The average plasma concentrations of olinciguat 1 h after dosing on Day 4 were 57.3 ± 38.7 nM (1 mg/kg), 132 ± 30 nM (3 mg/kg), and 245 ± 86 nM (10 mg/kg; each value represents the mean and SEM determined from 6 animals, n=3 Wistar, n=3 SHR).

### Dahl Salt-Sensitive Rat Model

#### Blood Pressure, Plasma Concentrations of Olinciguat, and Body Weight

**Figure 5:**
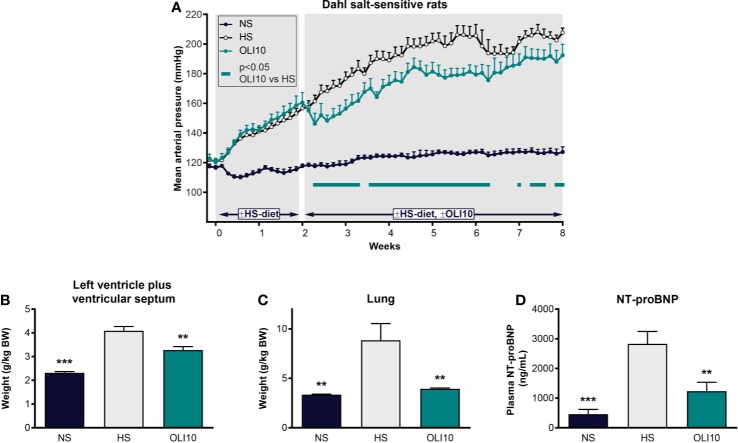
Effect of olinciguat on blood pressure, cardiac weights, lung weights, and NT-proBNP in Dahl salt-sensitive rat. **(A)** Mean arterial pressure vs time profiles in NS and HS controls, and HS rats treated with olinciguat 10 mg/kg (OLI10). HS groups were put on the HS diet starting Week-2. Olinciguat treatment was started on Week 0. Data are plotted as daily average MAP + SEM. **(B)** Weight of left ventricular wall plus ventricular septum normalized to BW. **(C)** Lung weight normalized to BW. **(D)** Plasma NT-proBNP. Data in **(B–D)** are plotted as mean + SEM, **p < 0.01, ***p < 0.001 vs HS control. For the OLI10 group, MAP measurements that were significantly (p < 0.05) lower than vehicle-treated rats are indicated by filled rectangles.

We next explored the effects of olinciguat treatment in the DSS rat model, which develops hypertension, heart failure, and renal dysfunction in response to a HS diet. At baseline, MAP was similar among all groups (121 ± 2 mmHg, **Figure 5A**). In the NS control group, MAP remained below 128 mmHg throughout the 2-week pretreatment and 6-week treatment periods. In the HS control group, MAP was significantly higher than in NS one day after high salt diet was initiated and increased to >155 mmHg at the end of the pretreatment period. MAP in HS steadily increased until the fourth week of the treatment period when it reached 206 ± 6 mmHg. The subsequent drop in MAP in HS appeared to be driven by decompensated heart failure in a subgroup as evidenced by low blood pressure, high NT-proBNP, and higher LV weight in these rats. Upon treatment initiation, MAP in OLI10 decreased from 161 ± 7 mmHg on day 14 to 146 ± 7 mmHg on day 16 and then steadily increased to 192 ± 7 mmHg, which was 15 mmHg lower than HS at the end of the treatment period. MAP in OLI10 was significantly lower than HS throughout most of the treatment period. Average body weights at the end of the treatment period were 460 ± 7, 364 ± 19, and 412 ± 11 g in NS, HS, and OLI10, respectively. Weights of animals in NS and OLI10 were significantly higher than HS. The average total plasma concentration of olinciguat was 25.4 ± 3.8 nM (n=6, at week 6).

#### LV Weight, Lung Weight, and NT-proBNP

To explore the impact of high salt diet and olinciguat on development of left ventricular hypertrophy, the weight of the LV+S, normalized to body weight, was compared across groups. At the end of the 6-week treatment period, LV+S weight was higher in HS (4.1 ± 0.2 g/kg BW) than in NS (2.3 ± 0.1 g/kg BW) (**Figure 5B**). LV+S was lower in OLI10 group (3.3 ± 0.1 g/kg BW) than HS. Cardiac weight was also lower in OLI10 than HS (data not shown).

Lung weight in HS (8.9 ± 1.7 g/kg BW) was 2.6-fold higher than in NS (3.3 ± 0.1 g/kg BW). However, lung weight in OLI10 was 4.0 ± 0.1 g/kg BW, only 1.2-fold higher than in NS, and significantly lower than HS (**Figure 5C**).

NT-proBNP in HS was 2,833 ± 416 pg/ml (n=7), 6.1-fold higher than in NS (458 ± 161 pg/ml [n=6]). NT-proBNP in OLI10 was 1,235 ± 301 pg/ml (n=6). NT-proBNP levels were lower in NS and OLI10 groups than in HS [**Figure 5D**].

#### Urine Protein Excretion and Kidney Weight

Twenty-four-h urine protein excretion (UPE) in HS was 10-fold higher than in NS (643 ± 60 mg vs 64.6 ± 6.7 mg). UPE in OLI10 was 599 ± 70 mg, which tended to be less than that in HS, yet the difference did not reach statistical significance. Normalized kidney weight was significantly greater in HS (10.9 ± 0.5 g/kg BW) than in NS (6.4 ± 0.14 g/kg BW); kidney weight in OLI10 (10.2 ± 0.23 g/kg BW) was not significantly different from HS.

### Post-myocardial Infarction Rat Model of Heart Failure with Reduced Ejection Fraction

The rat post-myocardial infarction (post-MI) model was studied as a model of heart failure with reduced ejection fraction. Eight weeks after MI, ejection fraction was lower in the MI control group (57.2 ± 4.0%) than in the Sham control group (77.4 ± 2.0%). Relative to MI controls, there was a trend (p=0.083) toward higher EF (67.9 ± 4.1%) in MI rats treated with 30 mg/kg/day olinciguat. Olinciguat plasma concentration at week 8 was 167 ± 11 nM.

### ZSF1 Rat Model of Metabolic Syndrome and Diabetic Nephropathy

ZSF1 rats, which are the F1 progeny of a cross between two rat strains that are heterozygous for mutant alleles of the leptin receptor gene (lean female Zucker diabetic [ZDF,+/fa] x male spontaneously hypertensive heart failure rats [SHHF,+/fa^cp^]), have been studied as a model of obesity and metabolic syndrome as well as diabetes and its complications (Tofovic et al., 2000; Bilan et al., 2011). We explored the effects of olinciguat in ZSF1 rats receiving background treatment with enalapril to mimic standard of care drug treatment for patients with diabetic kidney disease.

At week 10 of the treatment period, body weight was significantly higher in the obese control group (666 ± 17 g) than in the lean control group (511 ± 8 g); body weights in the treatment groups were not significantly different than in the obese control.

The average total plasma concentration of olinciguat in ENP+OLI30 at Week 10 was 510–610 nM.

#### Cardiovascular Effects

**Figure 6:**
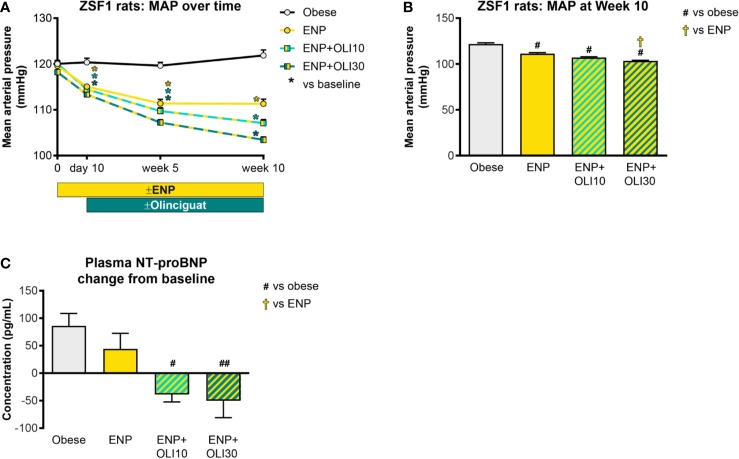
Effects of treatment on MAP and plasma NT-proBNP in the ZSF1 rat. **(A)** Timecourse of weekly average MAP throughout the study (*p < 0.05 vs baseline). **(B)** MAP at Week 10. **(C)** Change from baseline NT-proBNP. For **(B, C)**, ^#^p < 0.05 and ^##^p < 0.01 vs obese control, and ^†^p < 0.05 vs ENP. Data are plotted as mean ± SEM.

At baseline, mild hypertension was observed in all obese ZSF1 groups with average MAP in a range of 117–120 mmHg. At day 10, MAP in all groups receiving enalapril had decreased from baseline, and the reduction from baseline was maintained at week 5 and week 10 in all three treatment groups (**Figure 6A**). At Week 10 of the treatment period, MAP in all treatment groups was significantly lower than in the obese control group (122 ± 1 mmHg; **Figure 6B**). In addition, MAP in the ENP+OLI30 group (103 ± 1 mmHg) was significantly lower than the ENP control group (111 ± 1 mmHg). At week 10, MAP in ENP+OLI10 (107 ± 1 mmHg) trended lower (p=0.07) vs ENP.

Baseline plasma NT-proBNP levels were similar in all obese ZSF1 groups (193 ± 28, 209 ± 11, 148 ± 25, 226 ± 20 pg/ml in obese, ENP, ENP+OLI10, and ENP+OLI30, respectively). NT-proBNP, analyzed as change from baseline to the end of treatment, increased in the obese control (+86 ± 22 pg/ml) and ENP (+44 ± 28 pg/ml), and decreased in ENP + OLI10 (−39 ± 13 pg/ml) and ENP+OLI30 (−50 ± 31 pg/ml) groups (**Figure 6C**). Change from baseline NT-proBNP in the ENP+OLI10 and ENP+OLI30 groups was significantly different from that in the obese control, and there was a trend (p=0.058) toward a difference between ENP+OLI30 and ENP.

Heart weight in the obese control was 2.28 ± 0.03 g/kg BW. Heart weight was not affected in any of the treatment groups.

#### Renal Effects

**Figure 7:**
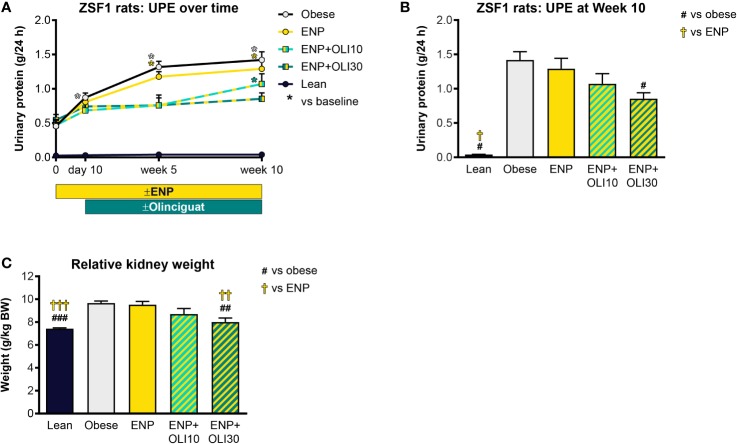
Effects of treatment on UPE and kidney weight in the ZSF1 rat. **(A)** Timecourse of UPE at baseline, Day 10 (end of pretreatment), Week 5, and Week 10 of the treatment period (*p < 0.05 vs baseline). **(B)** UPE at Week 10. **(C)** Relative kidney weight at end of treatment. For **(B, C)** ^#^p < 0.05, ^##^p < 0.01, ^###^p < 0.001 vs obese control, and ^†^p < 0.05, ^††^p < 0.01, ^†††^p < 0.001 vs ENP. Data are plotted as mean + SEM.

UPE was measured at various timepoints during the ZSF1 study. At baseline, UPE in the lean control was 0.026 ± 0.002 g/24h and did not change from baseline throughout the study (**Figure 7A**). Baseline UPE in the obese control (0.456 ± 0.055 g/24h) was 17.5-fold higher than in the lean control. Throughout the study, UPE in the obese control progressively increased, first achieving significance at week 5 (compared to baseline). UPE in ENP+OLI10 was only significantly different from its baseline at week 10. UPE in ENP+OLI30 did not significantly increase from its baseline at any time during the study. Furthermore, at week 10, UPE in ENP+OLI30 (0.853 ± 0.088 g/24h) was significantly lower than UPE in the obese control (1.42 ± 0.12 g/24h, **Figure 7B**).

At week 10, 24-h urine volume was 19.3-fold higher in the obese control (113.9 ± 17.7 ml) than the lean control (5.9 ± 0.5 ml), but urine volumes in the treated groups were not significantly different from the obese control (data not shown).

Total kidney weight was significantly greater in the obese control (9.7 ± 0.2 g/kg BW) and ENP (9.5 ± 0.3 g/kg BW) relative to the lean control (7.4 ± 0.07 g/kg BW). Kidney weight was lower in ENP+OLI30 (8.0 ± 0.4 g/kg BW) than in the obese control and ENP. Kidney weight in ENP+OLI10 (8.7 ± 0.5 g/kg BW) was numerically lower than the obese control and ENP (**Figure 7C**).

#### Renal Histology

**Figure 8:**
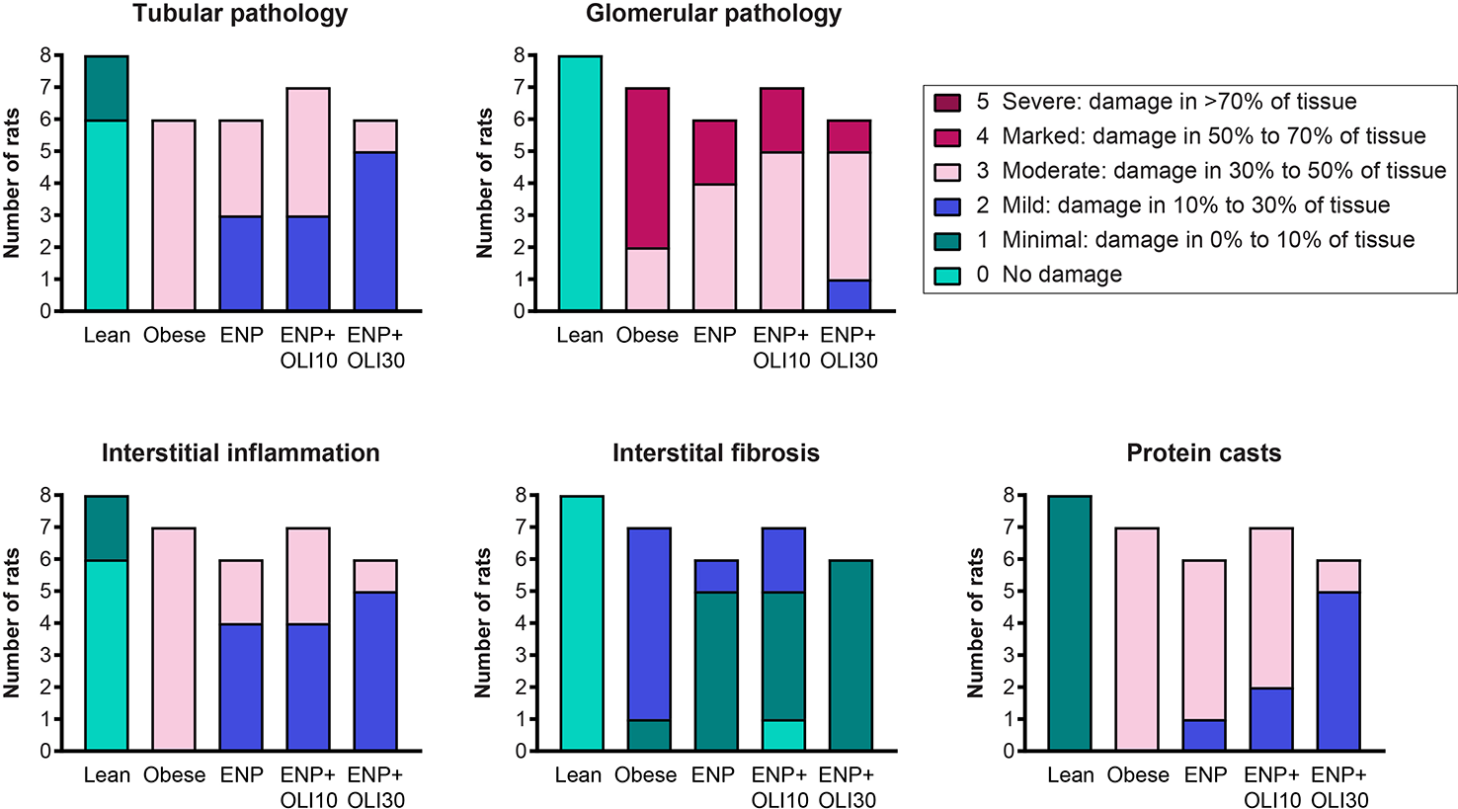
Stacked column plot displaying components of the renal histopathology score in the ZSF1 study. Colors indicate histopathology severity and the Y axis indicates number of rats at each level of severity.

Histological analysis of kidneys revealed that obese control rats developed severe glomerular pathology characterized by segmental mesangial expansion and hypercellularity, and moderate tubular pathology characterized by dilation, atrophy, attenuation of lining epithelium, and intratubular casts (**Supplemental Figure 1** and **Figure 8**). Glomeruli and tubules in obese control rats had moderate interstitial inflammation and mild interstitial fibrosis, mainly confined to areas of pathology. Obese control rats also had moderate protein casts within the renal tubules. The renal pathology sum score was significantly higher in the obese control group (14.6 ± 0.2) than in the lean control group (1.5 ± 0.3; p < 0.0001). The renal pathology sum scores in ENP (12.2 ± 0.4, p=0.025 vs. obese control) and ENP+OLI10 (12.1 ± 0.9, p=0.017 vs. obese control) were both significantly lower than the obese control. The renal pathology sum score in ENP+OLI30 (10.5 ± 0.6) was the lowest among all treatment groups, with the most robust p-value (p < 0.0001) vs the obese control. For each component of the renal pathology score there were fewer animals with more severe scores in the ENP group and even fewer in the ENP+OLI30 group (**Figure 8**).

#### Metabolic Effects

**Figure 9:**
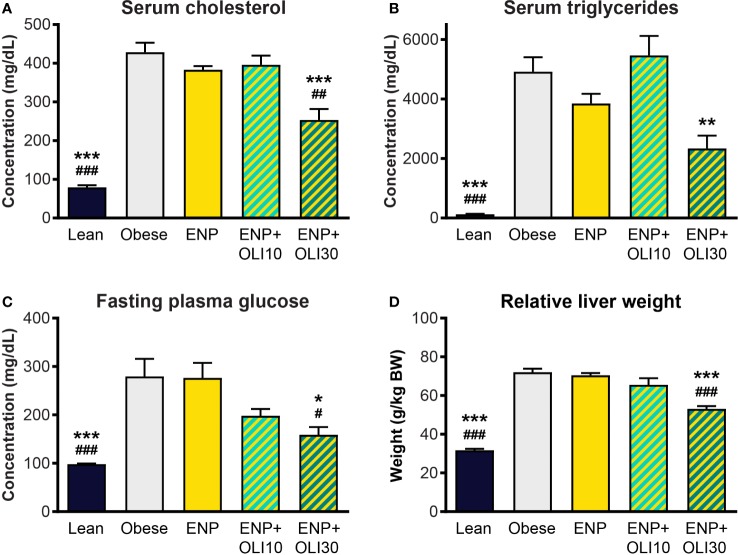
Effects of treatment on cholesterol, triglycerides, glucose, and liver weight in the ZSF1 rat model. * p < 0.05, ** p < 0.01, *** p < 0.001 vs obese control; # p < 0.05, ## p < 0.01, ### p < 0.001 vs ENP.

At week 10, serum cholesterol (428.6 ± 12.7 mg/dl) and triglycerides (4913 ± 487.1 mg/dl) were higher in the obese control than in the lean control (79.3 ± 5.7 mg/dl and 125.1 ± 17.1 mg/dl) (**Figures 9A, B**). Cholesterol and triglycerides in ENP were 383 ± 9.8 mg/dl and 3850 ± 326.0 mg/dl, respectively, which were not significantly different from the obese control. Cholesterol in ENP+OLI30 was 253.3 ± 28.5 mg/dl, 41% lower than that of the obese control and significantly lower than in both the obese control and ENP. Triglycerides in ENP+OLI30 were 2338 ± 433.9 mg/dl, 52% lower than in the obese control.

Fasting blood glucose in the obese control (279.4 ± 36.5 mg/dl) was significantly higher than in the lean control (98.1 ± 1.4 mg/dl) (**Figure 9C**). Glucose in ENP (276.5 ± 30.9 mg/dl) was not different from that in the obese control. Glucose in ENP+OLI30 (158.8 ± 16.1 mg/dl) was lower than in both the obese control and ENP. Glucose in ENP+OLI10 (198 ± 14.3 mg/dl) group tended to be lower than in the obese control, but this trend (p=0.092) was not significant.

Liver weight was significantly greater in the obese control (72.0 ± 1.9 g/kg BW) and ENP (70.5 ± 1.1 g/kg BW) than in the lean control (31.7 ± 0.7 g/kg BW). Liver weight was lower in ENP+OLI30 (53.1 ± 1.4 g/kg BW) than in the obese control and ENP. Liver weight in ENP+OLI10 (65.6 ± 3.4 g/kg BW) trended lower than the obese control and ENP group (**Figure 9D**).

### Mouse Model of Vascular Inflammation

**Figure 10:**
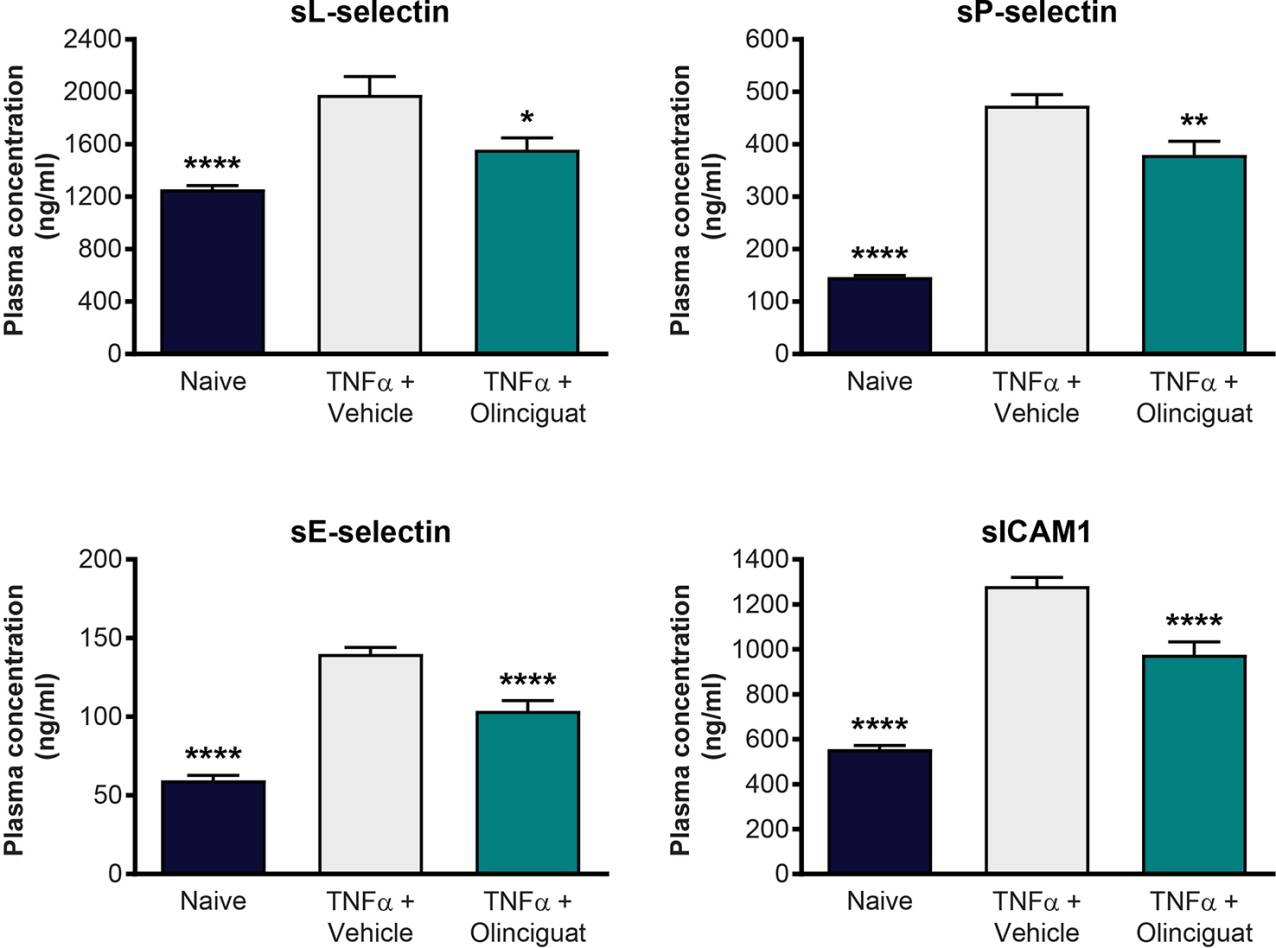
Effect of olinciguat on plasma levels of cellular adhesion molecules in mouse TNFα model of vascular inflammation. *p < 0.05, **p < 0.01, ****p < 0.0001 vs TNFα/vehicle control.

We explored the effect of olinciguat treatment on levels of soluble adhesion molecules arising from activated endothelium and leukocytes in a TNFα-induced model of vascular inflammation in mice. Compared to naïve controls, levels of plasma soluble adhesion molecules sL-selectin (1977 ± 141 vs 1,258 ± 27 ng/ml), sP-selectin (474 ± 21 vs 147 ± 3 ng/ml), sE-selectin (140 ± 4 vs 60 ± 3 ng/ml), and sICAM-1 (1,281 ± 39 vs 556 ± 17 ng/ml) were elevated in the TNFα/vehicle group of mice. As shown in **Figure 10**, plasma levels of sL-selectin (1559 ± 90 ng/ml), sP-selectin (380 ± 26 ng/ml), sE-selectin (104 ± 7 ng/ml), and sICAM-1 (976 ± 57 ng/ml) were lower in TNFα mice pretreated with 10 mg/kg olinciguat than in TNFα/vehicle controls. In the olinciguat-treated group, the plasma concentration of olinciguat was 544 ± 61 nM.

## Discussion

### Olinciguat Is a New sGC Stimulator

This is the first report of the pharmacologic properties of olinciguat, a member of the pyrazole-pyrimidine heterocyclic sGC stimulator class. Like other sGC stimulators, olinciguat is an NO-independent sGC agonist and it acts in synergy with NO to increase cGMP production by sGC (Mittendorf et al., 2009; Nakai et al., 2016; Tobin et al., 2018).

### Olinciguat Relaxed Vascular Smooth Muscle and Inhibited VSMC Proliferation

Pathological vascular remodeling is present in pulmonary arterial hypertension (PAH) as well as atherosclerosis and restenosis (Wang et al., 2018). All of the classes of drugs approved for the treatment of PAH, including PDE5 inhibitors and sGC stimulators, can affect pulmonary arterial tone, but are also believed to impact disease progression by inhibiting pathological vascular remodeling (Rubin, 2013). Olinciguat relaxed human vascular tissue with an EC_50_ in the nanomolar concentration range (24.3 nM). Importantly, olinciguat was a potent inhibitor of vascular smooth muscle proliferation (IC_50_ 29.6 nM), and this effect was potentiated 3× by the PDE5 inhibitor tadalafil. Interestingly, in our study tadalafil alone did not inhibit vascular smooth muscle cell proliferation at concentrations at or below 10 µM. Previously, it was reported that tadalafil inhibited proliferation of pulmonary artery smooth muscle from idiopathic pulmonary arterial hypertension (PAH) patients with an IC_50_ of 4.5 µM whereas it had no effect on pulmonary artery smooth muscle from healthy subjects (Yamamura et al., 2017). Our *in vitro* results suggest that direct stimulation of sGC may have more potent vascular anti-remodeling effects than PDE5 inhibition, and that the effects of sGC stimulation may be enhanced by PDE5 inhibition. The hypothesis that tadalafil may enhance anti-remodeling effects of olinciguat should be tested in an *in vivo* model characterized by vascular remodeling, such as the monocrotaline or Sugen/hypoxia model of pulmonary hypertension. Exploration of potential synergies between sGC stimulators and PDE5 inhibitors in patients has been precluded due to a contraindication based on a small study with riociguat and sildenafil in patients with PAH (Galie et al., 2015).

### Olinciguat Is Orally Available and Primarily Cleared by the Liver in Rats

In pharmacokinetic studies in rats, olinciguat exhibited low to moderate clearance and 40% oral bioavailability. We expected the pharmacokinetic profile in rats to translate to once-daily oral dosing in humans. Indeed, in healthy human subjects, after repeat dosing with orally administered tablets, olinciguat had a long terminal-phase half-life (~30 h) and a correspondingly low peak-to-trough plasma ratio (Mittleman et al., 2017). It is anticipated that this profile will translate to consistent pharmacodynamic effects from one daily dose to the next. In mass balance studies in rats, most (> 95%) of the radiolabeled olinciguat dose was recovered in feces, indicating predominantly hepatic clearance and minimal renal clearance. Likewise, renal clearance was negligible (≤0.3% of apparent total body clearance [CL/F]) in healthy human subjects. The low renal clearance of olinciguat may enable its use in patients with renal impairment without the need for dose adjustment.

### Olinciguat Evenly Distributed Between Vascular Compartment and Tissues

In male and female rats, olinciguat had a mean pharmacokinetic volume of distribution of 1.0 L/kg, indicating similar partitioning between plasma and tissues. Consistent with this finding, whole body autoradiography studies revealed that tissue-to-plasma ratios of olinciguat in most tissues were between 0.5 and 2.0, including heart, lung, skeletal muscle, vascular tissue, and adipose. Organs containing the highest levels of [^14^C]-olinciguat equivalents included the highly perfused liver and kidneys; organs with the lowest levels included CNS tissues and bone. In contrast, praliciguat, another pyrazole-pyrimidine heterocyclic sGC stimulator in clinical development has a very high volume of distribution (10.5 L/kg) and extensive distribution to tissues (Tobin et al., 2018). The even distribution of olinciguat between plasma and organs suggests the potential for balanced vascular effects (restoration of endothelial function, inhibition of vascular inflammation and vascular smooth muscle proliferative remodeling, effects on hematocytes), and extravascular effects (attenuation of organ fibrosis and inflammation).

### Olinciguat Affected Hemodynamics in Normotensive and Hypertensive Rats

Given olinciguat's activity as an sGC stimulator and vasodilator *in vitro*, and the fact that drugs targeting the NO-sGC-cGMP pathway typically reduce blood pressure, we explored olinciguat's effects on hemodynamics in normotensive Wistar and spontaneously hypertensive rats. In both strains of rats, dose-related reductions in MAP and increases in heart rate were observed, and the largest effects were observed in the first 6 h after dosing, corresponding to the time period when plasma levels of olinciguat were highest. Effects on heart rate appeared more pronounced in normotensive rats. In contrast, larger reductions in MAP were observed in hypertensive rats. The stronger reflex tachycardia response in normotensive rats relative to hypertensive rats is expected since baroreceptor reflex control of heart rate is known to be reduced in both hypertensive rats and humans (Mancia et al., 1978). At 10 mg/kg/day in the hypertensive DSS rat and at 30 mg/kg/day in the mildly hypertensive ZSF1 rat model, olinciguat treatment was associated with lower blood pressure. The relatively small effect on BP in hypertensive DSS rats may be explained by the low olinciguat plasma levels attained in that model (**Table 2**), and the small effect in the ZSF1 model may in part be explained by the fact that these rats develop only mild hypertension and olinciguat was studied in combination with enalapril (which itself lowered blood pressure significantly). In both the DSS model and the ZSF1 model, improvements in relevant pathologies were observed at doses which had only small effects on blood pressure.

**Table 2:** Pharmacological effects of olinciguat in preclinical models.

Model	Olinciguat dose (mg/kg or mg/kg/day)	Olinciguat concentration (free)^z^	Blood pressure (mmHg)	Pharmacological effects
Human cellular sGC assay (HEK-293) EC_50_	n/a	42.7–105 nM	n/a	n/a
Rat vascular smooth muscle proliferation EC_50_	n/a	22.4–39 nM	n/a	n/a
Human vascular relaxation EC_50_	n/a	16–38 nM	n/a	n/a
Normotensive rat	10	1.6–3.3 nM^a,e^	−11^b^	↑ ΔHR 134 BPM
Spontaneously hypertensive rat	10	1.6–3.3 nM^a,e^	−26^b^	↑ ΔHR 86.2 BPM
DSS rat hypertension & heart failure	10	0.22–0.29 nM^e^	−16^c^	↓ cardiac hypertrophy ↓ lung weight ↓ NT-proBNP
Post-MI rat heart failure	30	1.6–1.8 nM^e^	n.d.	↑ ejection fraction (trend)
ZSF1 rat metabolic syndrome and diabetic nephropathy	30	5.1–6.1 nM^e^	−8^d^	↓glucose ↓ cholesterol ↓ triglycerides ↓ liver weight ↓ proteinuria ↓ kidney weight ↓ renal histopath
TNFα mouse vascular inflammation model	10	8.2–10.2 nM^f^	n.d.	↓ sL-selectin ↓ sP-selectin ↓ sE-selectin ↓ sICAM-1

^a^Concentration determined from pooled samples from Wistar and SHR. ^b^Maximum absolute change in MAP. ^c^Difference in MAP between olinciguat-treated and disease control at end of treatment period. ^d^Difference in MAP at end of treatment between enalapril+olinciguat 30 mg/kg and enalapril monotherapy groups. ^e^Free concentration in rat studies determined by multiplying total plasma concentration by 1.0% free determined in a rat equilibrium plasma protein binding study. ^f^Free concentration in mouse determined by multiplying total concentration in plasma by 1.7% free, determined in a mouse equilibrium plasma protein binding assay.

### Cardioprotective Effects of Olinciguat

Drugs targeting the NO-sGC-cGMP pathway have a long history of use in cardiac conditions. Nitroglycerin, an NO donor and coronary artery vasodilator, has been used to treat angina for more than a century. Intravenous nitroglycerin is also commonly used in acute heart failure to provide rapid dyspnea relief. Relevant mechanisms of nitroglycerin in acute heart failure include reducing myocardial strain, and hemodynamic benefits resulting in decreased right ventricular and left ventricular filling pressures (Alzahri et al., 2016). By enhancing effects of NO-mediated signaling, an sGC stimulator might also be expected to favorably affect the heart.

The cardioprotective effects of olinciguat were tested in two models of heart failure: the rat post-MI model of heart failure with reduced ejection fraction, and the DSS hypertension model, which develops heart failure with preserved ejection fraction. In the post-MI model, olinciguat treatment attenuated the reduction in ejection fraction (67.9%) relative to the MI group, which had a more pronounced reduction in ejection fraction (57.2%). In this post-MI study, blood pressure was not monitored and only one dose level of olinciguat was tested. A dose-response study in telemetered MI rats could provide a more complete understanding. In the DSS model, olinciguat treatment was associated with lower left ventricular hypertrophy and lower levels of the cardiac strain marker NT-proBNP (56% lower than untreated controls). This is the first report of the effects of sGC stimulation on lung weights in the DSS model, which, coupled with our hemodynamic, and cardiorenal data, add to a growing understanding of the effects of sGC stimulation in the DSS model (Geschka et al., 2011). Relative to NS rats, lung weight was markedly (2.6×) increased in HS DSS rats, which may have been a consequence of pulmonary congestion due to volume expansion, impaired LV function, or both. In olinciguat-treated rats however, lung weight was only 10% greater than in NS rats. The fact that lung weight in HS rats treated with olinciguat does not appear to increase suggests that olinciguat may reduce pulmonary congestion; in humans, this effect would reduce dyspnea, a major driver of hospitalizations in patients with heart failure. The cardiac effects in DSS rats were observed at an olinciguat dose that was associated with a modest reduction in blood pressure. Given that the effect of 10 mg/kg/day olinciguat on blood pressure was modest, that olinciguat-treated rats remained hypertensive, and that a 30 mg/kg/day dose of olinciguat in the ZSF1 model was associated with greater renal benefit than the 10 mg/kg/day dose, one might expect more profound cardiac effects of olinciguat in the DSS model at a higher dose.

Aged ZSF1 rats have been reported to develop heart failure consistent with HFpEF (Franssen et al., 2016; Salah et al., 2018). In our ZSF1 study, NT-proBNP, a marker of cardiac strain and heart failure severity, increased from baseline to the end of treatment in the obese and enalapril monotherapy groups. In contrast, NT-proBNP decreased in the groups of rats treated with olinciguat. However, cardiac weights in all treatment groups were not different from the obese control. Although our study was not designed to explore heart failure, olinciguat's effect on NT-proBNP was encouraging.

The cardioprotective effects of olinciguat are consistent with effects previously reported for other pharmacological agents in the NO-sGC-cGMP pathway, including nitrates, sGC stimulators and activators, and PDE5 inhibitors (Park et al., 2018). As cardiac functional impairment and remodeling are associated with a variety of diseases such as heart failure, pulmonary hypertension, sickle cell disease, and muscular dystrophy, the cardioprotective effects of olinciguat may have the potential to provide therapeutic benefit in a wide range of diseases.

### Olinciguat Treatment Affected Cardiometabolic Risk Factors and Liver Weight

Hyperglycemia and hyperlipidemia are risk factors for cardiovascular disease, the leading cause of death for patients with diabetes (Haffner and Cassells, 2003; Stone et al., 2013). We studied the effects of olinciguat in ZSF1 rats, which develop obesity, metabolic syndrome, and diabetes and its complications. Glucose, cholesterol, and triglycerides were higher in obese ZSF1 rats than in lean controls. Remarkably, treatment with olinciguat was associated with significantly lower glucose (↓43%), cholesterol (↓41%), and triglycerides (↓52%). Although the mechanism is not completely understood, our data add to a growing body of evidence that pharmacological stimulation of sGC may have favorable effects on metabolism in obesity and metabolic syndrome. In 2015, it was reported that the sGC stimulator BAY 41-8543 protected against weight gain and improved the diabetic phenotype in a diet-induced obesity mouse model (Hoffmann et al., 2015), and in a study in 26 patients with diabetes and hypertension, 14 days of treatment with the sGC stimulator praliciguat reduced fasting glucose level and total and LDL cholesterol levels (Hanrahan et al., 2020).

Although the obese ZSF1 rat is not a model of liver disease per se, this model is known to develop steatosis with age, typically beginning around 20–24 weeks (Zambad et al., 2011; Borges Canha et al., 2017). As a model of diabetes, disease progression may reflect that seen in humans with type 2 diabetes who develop increasingly severe comorbidities. In our study, greater liver weight in obese ZSF1 rats (130% greater than lean control) at 26 weeks may have been due to steatosis. The lower liver weight in olinciguat-treated rats may derive from potential antisteatotic effects (supported by olinciguat's effects on glucose, cholesterol, and triglycerides in this model) and/or its effect on right heart function (supported by its effect on NT-proBNP levels in this model).

### Renoprotective Effects of Olinciguat

The progressive nephropathy and associated proteinuria that develops in the obese ZSF1 rat mirrors the progressive nature of human diabetic kidney disease (Dower et al., 2017). In a previous study, 8-week old obese ZSF1 rats treated with a high dose of enalapril (60 mg/kg/day in drinking water for 24 weeks) had a profound reduction in MAP (~50 mmHg). Albuminuria, interstitial fibrosis, and glomerulosclerosis were nearly eliminated in the enalapril-treated rats, but glucose homeostasis was not improved (Bilan et al., 2011).

We explored the effects of olinciguat in ZSF1 rats receiving 3 mg/kg/day enalapril to model a clinically relevant dose of the standard of care in patients with diabetic kidney disease. In our study, obese controls exhibited mild hypertension and, in addition, developed progressive proteinuria, which increased by 153% over the 10-week treatment period. Treatment with enalapril alone did not attenuate proteinuria progression (168% increase over 10-week treatment period), despite lowering blood pressure. However, the addition of 30 mg/kg/day olinciguat did attenuate progression of proteinuria (limited to a 56% increase over 10 weeks). A limitation of the study is that we only included one dose level within the clinically relevant range for enalapril.

Renal hypertrophy has been associated with diabetes and diabetic nephropathy in humans and is a common feature in animal models of chronic kidney disease. Hypertrophy may arise because of changes in tubular function aimed at preventing loss of water in electrolytes in response to altered renal hemodynamics. Hypertrophy may also arise as a consequence of hyperplasia and hypertrophy of the nephron caused by hyperglycemia and hyperglycemia-induced overproduction of growth factors and cytokines (Fine, 1986; Wolf and Ziyadeh, 1999). Kidney weights in obese ZSF1 and obese ZSF1 treated with enalapril were 31% and 28% greater than in the lean control group, indicating development of renal hypertrophy in these groups. However, ZSF1 rats treated with olinciguat 30 mg/kg/day plus enalapril did not develop renal hypertrophy.

Consistent with the effect on urine protein and renal hypertrophy, olinciguat 30 mg/kg/day plus enalapril significantly improved the composite renal histopathological score in ZSF1 rats, whereas enalapril alone did not have a significant effect.

Blood pressure in enalapril-treated obese ZSF1 was lower than in obese controls, and olinciguat in combination with enalapril produced a small but significant reduction in blood pressure beyond that produced by enalapril alone. The effect of olinciguat on blood pressure indicates sGC target engagement and may account for some of the favorable effects of olinciguat on the kidney in this model. However, the pathogenesis of nephropathy in the ZSF1 model is not primarily driven by hypertension and may instead be a consequence of metabolic disturbances that make the glomerulus susceptible to injury under normal glomerular pressures (Griffin et al., 2007). It is therefore more likely that the renoprotective effects of olinciguat were due to direct effects of olinciguat on the kidney. A renal function and hemodynamic study of olinciguat monotherapy at a dose devoid of effects on systemic blood pressure may add to mechanistic understanding.

In the DSS model, which developed proteinuria and kidney hypertrophy in addition to chronic hypertension, 10 mg/kg/day olinciguat treatment was associated with mild reductions in proteinuria and kidney weights, but these effects were not significant. Although blood pressure was lower in the olinciguat-treated group than in controls, hypertension remained severe in olinciguat-treated rats (MAP 192 mmHg) by the end of the treatment period. In the face of this chronic hypertensive insult, the direct renoprotective effects of olinciguat may have been insufficient to prevent the development of the renal pathology. We speculate that a higher dose of olinciguat may reduce the pressure insult on the kidney and at the same time maximize its direct renoprotective actions. The renoprotective effects of olinciguat are consistent with pharmacologic studies and mechanistic studies that have been reported for NO-cGMP pathway modulators (reviewed in (Stasch et al., 2015)).

### Olinciguat Effects on Vascular Inflammation

At sites of inflammation or vascular injury, accumulation of activated leukocytes in the affected tissue promotes inflammation, which can lead to compromised endothelial barrier function and tissue damage. An initiating step in this process is leukocyte and endothelial cell activation, resulting in cell surface expression of leukocyte and endothelial cell adhesion receptors, that facilitate the adhesion of leukocytes to the vascular wall and their recruitment to the affected tissue. In sickle cell disease, which has been characterized as a disease of hemolysis-induced NO insufficiency, local vasoconstriction and adhesion of leukocytes and sickled red blood cells to the vascular endothelium can trigger painful vaso-occlusive crisis. Inhibition of NO-cGMP signaling promotes endothelial activation, leukocyte adhesion, and extravasation (Hossain et al., 2012). In contrast, stimulation of NO-cGMP signaling attenuates the expression of adhesion molecules and reduces leukocyte-endothelial cell interactions (Kubes et al., 1991; Ahluwalia et al., 2004). Using a TNFα model of inflammation in mice, we sought to understand whether olinciguat would have an effect on endothelium- and leukocyte-derived soluble adhesion molecules. Levels of plasma biomarkers of endothelial activation (sP-selectin, sE-selectin, and sICAM1) and leukocyte activation (sL-selectin) were lower in olinciguat-pretreated mice than in controls, which had significantly increased plasma levels of these markers as compared to naïve animals. These data suggest that olinciguat treatment may attenuate leukocyte and vascular endothelial cell activation exacerbated by inflammation and NO insufficiency and thereby reduce cellular adhesion in the vasculature. This effect combined with its potential to enhance NO-mediated vasodilation at sites of endothelial dysfunction suggest that olinciguat holds promise as a potential treatment to prevent vaso-occlusive pain and protect against leukocyte-mediated tissue damage in hematological disorders including sickle cell disease.

### Highlights and Looking Forward

In summary, this is the first report of the pharmacokinetics, mass balance, and broad pharmacological characterization of the clinical-stage sGC stimulator olinciguat*. In vitro* data suggest that olinciguat may be more effective than the PDE5 inhibitor tadalafil in preventing pathological vascular remodeling and that tadalafil may potentiate its activity. Olinciguat affected hemodynamics in hypertensive and normotensive rats. Olinciguat demonstrated cardioprotective potential in cardiac impairment models of different etiologies. In a rat model of diabetes and metabolic syndrome that progresses to diabetic nephropathy, olinciguat treatment was associated not only with large reductions in glucose, cholesterol, and triglycerides—all cardiovascular risk factors—but also with renoprotective effects. In a mouse model of vascular inflammation, olinciguat treatment was associated with reduced levels of endothelial and leukocyte-derived soluble adhesion molecules in plasma. Olinciguat was orally available in rats, distributed evenly between blood and most tissues, and was primarily cleared by the liver. The PK and pharmacological features reported here suggest that olinciguat may have substantial therapeutic potential particularly optimized for diseases where its balanced vascular and extravascular distribution can provide broad, multiorgan benefits. Olinciguat's profile suggests therapeutic potential in the two forms of pulmonary hypertension for which riociguat, the only marketed sGC stimulator, is currently indicated. Olinciguat's PK (supportive of QD dosing), tissue distribution, and predominant hepatic clearance differentiates it from other sGC stimulators with respect to dosing frequency and target organ efficacy.

## Data Availability Statement

The datasets for this manuscript are not publicly available because of their proprietary nature. Requests to access the datasets should be directed to jjones@cyclerion.com.

## Ethics Statement

The animal study was reviewed and approved by Institutional Animal Care and Use Committee. Human tissues were obtained from donors with proper authorization and full ethical approval (ReproCELL Europe Ltd., Beltsville, MD). The collection and use of tissue was approved by the West of Scotland Research Ethics Committee (12/WS/0069) and all participating donors provided informed written consent prior to the surgical procedure.

## Author Contributions

DZ, CS, JT, BT, PG, KS, AB, SJ, SB, RS, AC, Y-TC, RG, GM, MC, and JM were involved in experiment conception and design. CS, JT, BT, PG, KS, AB, SJ, SB, RS, AC, and JM executed experiments. DZ, CS, JT, BT, PG, KS, AB, SJ, SB, RS, AC, JJ, and JM analyzed data. DZ, CS, JT, BT, PG, KS, AB, JW, GP, JC, GM, and JM provided data interpretation. DZ, CS, JT, BT, AB, JJ, GP, JC, and JM drafted text. DZ, CS, JT, KS, Y-TC, RG, EB, JJ, JW, GP, JC, GM, MC, and JM revised critically for important intellectual content.

## Conflict of Interest

All authors are current or former employees of Ironwood Pharmaceuticals or Cyclerion Therapeutics which was spun out of Ironwood Pharmaceuticals on April 1, 2019.

This research was funded by Cyclerion Therapeutics. Scientists at Cyclerion were responsible for study design, collection, analysis, and interpretation of the results.
