# Supplementary material for: Olinciguat, an Oral sGC Stimulator, Exhibits Diverse Pharmacology Across Preclinical Models of Cardiovascular, Metabolic, Renal, and Inflammatory Disease
Source: Front Pharmacol. 2020 Apr 8;11:419. doi: 10.3389/fphar.2020.00419 (PMC7156612; doi:10.3389/fphar.2020.00419)

# Supplemental Materials

## Rat whole body autoradiography

Olinciguat-derived ^14^C was determined in the following tissues: adipose (brown and white), adrenal gland, aorta, vena cava, bile (in duct), blood (cardiac), bone, bone marrow, brain (cerebrum, cerebellum, hippocampus, medulla), cecum (and contents), epididymis, esophagus, eye (uvea and lens), Harderian gland, heart, kidney (cortex and medulla), large intestine (and contents), liver, lung, lymph node, mammary gland region, oral mucosa, pancreas, penis, pituitary gland, prostate gland, salivary gland, seminal vesicles, skeletal muscle, skin (pigmented and non-pigmented, small intestine (and contents), stomach (gastric mucosa and contents), spleen, spinal cord, testis, thymus, thyroid, and urinary bladder (and contents).

## Supplemental Figure 1

Representative images of tubulo-interstitial (H&E) and glomerular (PAS) pathology in ZSF1 lean, obese, ENP and ENP+OLI30 rats. Renal morphology was normal in lean control rats. On H&E sections, obese control rats developed tubular dilation (black arrows), intra-tubular protein casts (green arrows) and interstitial inflammation. On PAS sections, the glomeruli of obese control rats were moderately enlarged; in addition,a segmental increase in PAS-positive mesangial matrix (blue arrows) and moderate to marked hypercellularity of the tuft were observed. Overall, glomerular and tubulo-interstitial pathology appeared less pronounced in ENP+OLI30 treated rats than in obese controls.

**
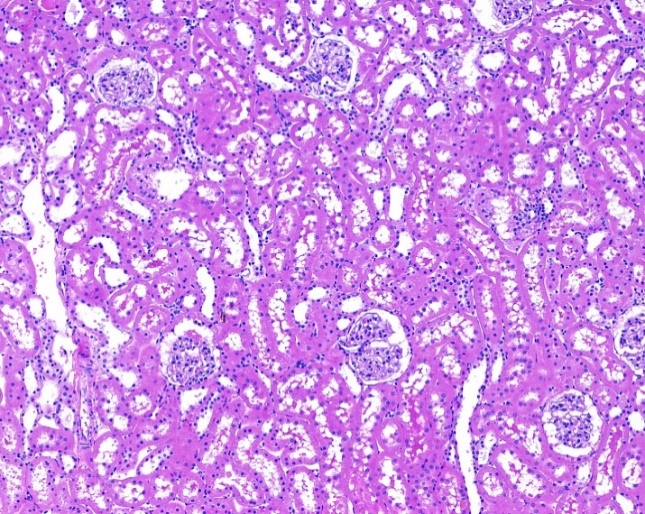
Supplemental Figure 1**


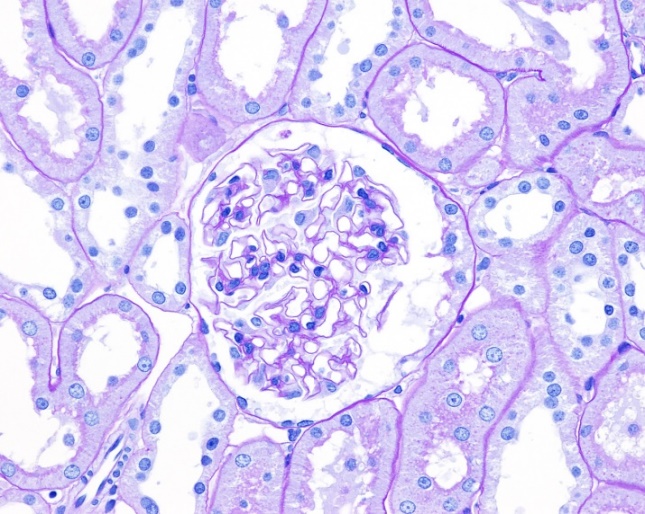


H&E

Obese

Lean

PAS


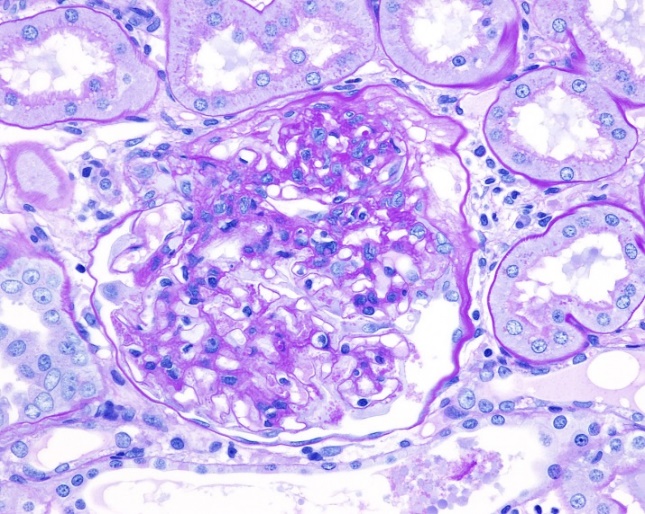

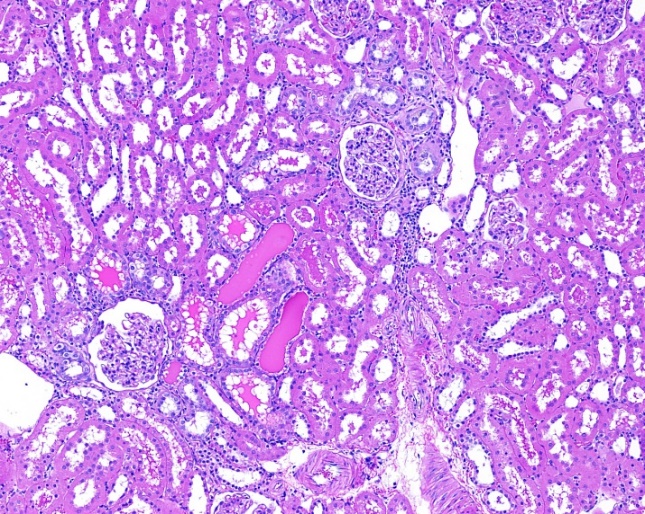

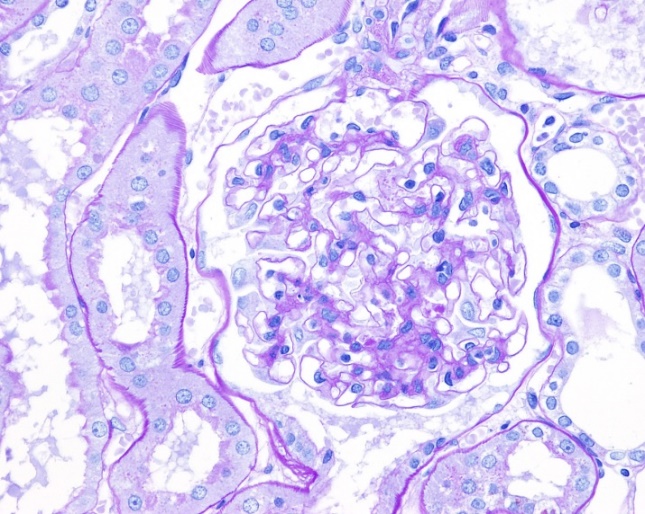


ENP

ENP + OLI30


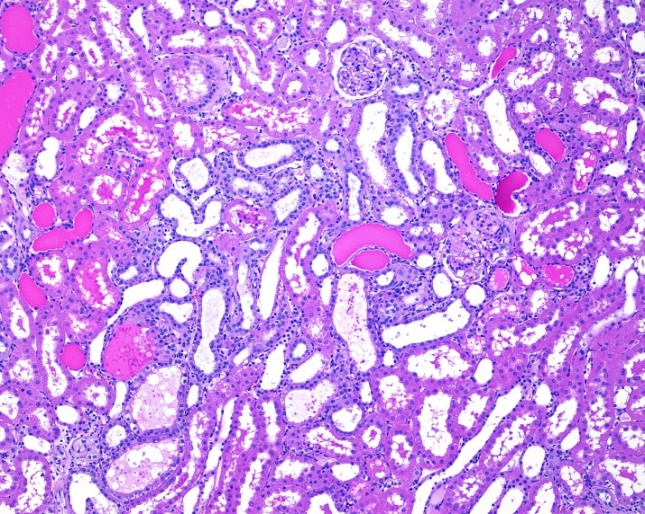

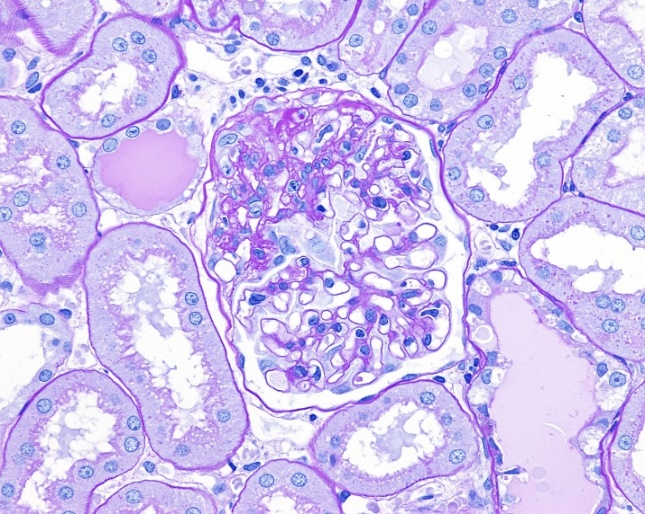

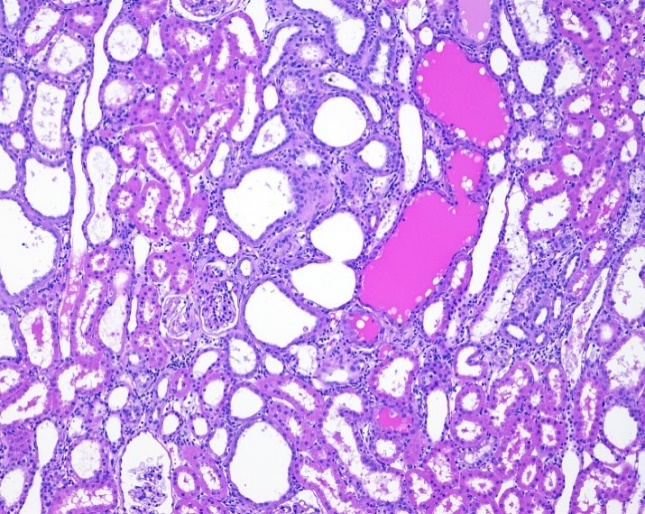

Supplement: Supplementary file 1 [file DataSheet_1.docx]
